# Hybrid statistical‐machine learning approach for analyzing legacy and new phosphorus losses from subsurface drainage systems

**DOI:** 10.1002/jeq2.70145

**Published:** 2026-02-13

**Authors:** Emeka Aniekwensi, Ehsan Ghane

**Affiliations:** ^1^ Department of Biosystems and Agricultural Engineering Michigan State University East Lansing Michigan USA

## Abstract

Phosphorus (P) is essential for crop growth but leaches through subsurface drainage discharge, impacting water quality. This study's objectives are to (1) apply hybrid statistical‐machine learning to quantify the contributions of incidental (new) and legacy (old) P in drainage discharge from organic site and inorganic site and (2) evaluate the effect of manure application timing on P loss. We collected data from two on‐farm sites in southeast Michigan, USA. A linear regression equation was used to analyze P load based on drainage discharge and fertilizer application timing. The data were split into calibration and validation sets, and machine learning was used for training. The results showed strong model prediction performance. Organic fertilizers contributed approximately twice the observed total phosphorus (TP) loss (7.54 kg ha^−^^1^ vs. 3.73 kg ha^−^^1^) and nearly four times the dissolved reactive phosphorus (DRP) loss (4.90 kg ha^−^^1^ vs. 1.05 kg ha^−^^1^) compared to inorganic P loss, mainly due to the greater P application rate and higher soil test P. When applied during winter months (December–January), organic fertilizer contributed to greater new P loss, whereas early fall applications (October–November) resulted in lower new P loss, showing the importance of application timing. At the organic site, legacy P was the dominant contributor to TP and DRP losses, accounting for 84% and 79% of losses, respectively. At the inorganic site, legacy P was responsible for 97% of TP loss and the entirety (100%) of DRP loss. In conclusion, legacy P was the dominant source of P loss through drainage discharge, and winter organic fertilizer application significantly increased new P loss.

## Plain Language Summary

Phosphorus helps crops grow but can pollute water when it drains from fields. Most losses came from legacy phosphorus already in the soil. Winter applications of organic fertilizer caused much higher new phosphorus loss than fall applications. The timing of fertilizer application considerably influences phosphorus loss, underscoring the need for improved management practices to minimize nutrient loss.

**Abbreviations**
DRP: dissolve reactive phosphorus
KGE: Kling–Gupta efficiency
NSE: Nash–Sutcliffe efficiency
PBias: percent‐bias
TP: total phosphorus

## 1 INTRODUCTION

Eutrophication is a major environmental challenge affecting freshwater systems worldwide, including the Midwest United States and the Great Lakes. This phenomenon depletes oxygen levels, endangers aquatic life, and increases the cost of water treatment. The annual economic burden, encompassing recreation, real estate devaluation, species protection, and water purification, amounts to approximately $2.2 billion (Dodds et al., 2009).

Annual fertilizer application boosts crop yield but often ra ises phosphorus (P) levels in subsurface drainage after application. This rise in P concentration is the sum of the existing P in the soil (old or legacy P) and the newly added P in fertilizer (new or incidental P; Osterholz et al., 2023). The distinction between “new” and “old” P in drainage discharge was first proposed by Osterholz et al. (2023). Using a linear regression model, the authors assessed the contribution of new P to P loss in drainage discharge across eight on‐farm sites in Ohio and found that new P accounted for 0%–17% of the annual dissolved reactive phosphorus (DRP) loss. In a subsequent study, Osterholz et al. (2024) expanded their analysis to 38 on‐farm sites in Ohio and Indiana, reporting new P contributions of 14% to DRP and 5% to total phosphorus (TP) in drainage discharge. Despite these two studies on new P in northwest Ohio, more research is needed in other regions using alternative methods to improve our understanding of P dynamics. Other methods to distinguish new versus legacy soil P, such as oxygen isotope tracers (Ishida et al., 2024; Liu et al., 2023; Tye et al., 2021), require complex, costly lab work. Our study used machine learning–based sensitivity analysis to predict new P contributions offering a more efficient and scalable alternative.

Machine learning algorithms can be used as an alternative method to determine the regression coefficients of the regression equation. Thus far, there have been no other studies that have used machine learning algorithms to develop a regression model for determining new P loss. Furthermore, Dialameh and Ghane (2023) showed rapid fluctuations of P concentration associated with varying flow rates in drainage discharge, underscoring the challenges in accurately predicting P loss and emphasizing the importance of evaluating machine learning algorithms for capturing such dynamic patterns in new P loss. Machine learning has been successfully applied across diverse aspects of biosystems engineering and agriculture, including crop yield prediction (Jeong et al., 2016), soil nutrient estimation (Gurubaran et al., 2023), precision irrigation scheduling (Benos et al., 2021), and environmental impact assessment (Liakos et al., 2018). In these applications, machine learning has consistently demonstrated high predictive accuracy and adaptability, making it a promising tool for modeling. There is a need to determine new P contribution in drainage discharge using a hybrid statistical‐machine learning model.

Previous research has indicated that the rate, timing, and placement of fertilizers can significantly affect P loss in fields in Ohio and Indiana (Dialameh & Ghane, 2023; Hanrahan et al., 2019; Osterholz et al., 2024). Hanrahan et al. (2019) further emphasized that the fertilizer source is crucial for P loss. Their research differentiated the P loss impact of organic versus inorganic fertilizers, stating that organic fertilizers result in higher P concentrations than inorganic ones. Dialameh and Ghane (2023) and Schroeder et al. (2004) found that event flow accounts for over 59% of TP loss. Osterholz et al. (2024) also observed a sharp increase in TP concentration when events occurred soon after manure or fertilizer application. Their study showed that late fall P application increased the risk of new P loss through subsurface discharge. However, the effect of manure timing on P loss in a colder climate such as that in Michigan has not been investigated. Therefore, there is a need to evaluate the effect of manure timing in other locations.

A review of the literature reveals a need to identify the sources of P (legacy or new) at other locations, as few publications have determined these sources in Ohio and Indiana (Osterholz et al., 2024). Therefore, the objectives of this study are to (1) assess the contribution of new P loss from organic and inorganic fertilizer sources through drainage discharge using hybrid statistical‐machine learning and (2) evaluate the implication of manure application timing on new P loss. The value of this study is to provide new insight into new P contributions in a cold climate and inform policymakers on when nutrient reduction strategies should be prioritized.

## 2 MATERIALS AND METHODS

### 2.1 Site description

**FIGURE 1:**
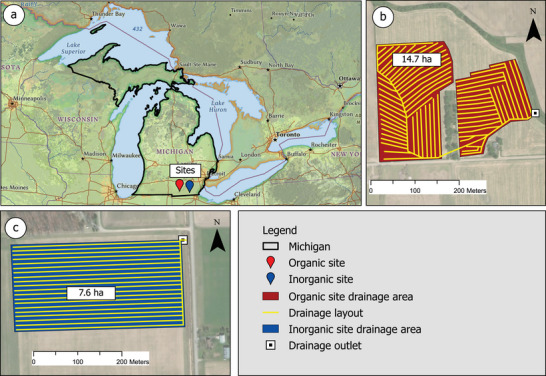
(a) Locations of the on‐farm experimental sites in Michigan, USA. (b) Drainage layout of the organic site. (c) Drainage layout of the inorganic site.

The experiment took place on two privately owned on‐farm research sites: organic site and inorganic site, located within a 15.6‐km radius of each other (Figure 1). The organic and inorganic farm sites were under free drainage and had areas of 14.7 and 7.6 ha, respectively. The data collection commenced on January 1, 2019, and concluded on December 31, 2024, spanning 6 years. The organic site features Blount loam soil, which is somewhat poorly drained soil under natural conditions, whereas the inorganic site consists of Ziegenfuss clay loam, which is a poorly drained soil under natural conditions (Soil Survey Staff, 2025). Detailed soil physical properties of the sites can be found in Table S1. The organic site had an average drain depth of 0.90 m and a spacing of 13.0 m, whereas the inorganic site had an average drain depth of 0.82 m and a spacing of 10.05 m. The cropping system was a corn–soybean rotation. The inorganic site received inorganic fertilizers, and the organic site received both organic and inorganic fertilizers, as summarized in Table 1.

**TABLE 1:** Phosphorus fertilizer application date, type, and rate throughout the experimental period.

Site	P application date	P application rate (kg ha^−1^)	Form of P fertilizer	Application method
Organic	January 14, 2019	43.2	Solid organic	Surface broadcast
	December 5, 2019	5.3	Liquid organic	Surface broadcast
	May 27, 2020	3.0	Liquid inorganic	Starter fertilizer in‐furrow
	October 1, 2020	3.4	Liquid organic	Surface broadcast
	January 5, 2022	90.5	Liquid organic	Surface broadcast
	October 10, 2022	22.3	Liquid organic	Surface broadcast
	November 13, 2023	24.9	Liquid organic	Surface broadcast
	**Average annual**	**48.2**		
Inorganic	April 9, 2019	22.5	Solid inorganic	Surface broadcast
	May 12, 2020	9.5	Liquid inorganic	2 × 2 banded injection
	May 14, 2023	38.0	Liquid inorganic	2 × 2 banded injection
	**Average annual**	**23.2**		

*Note*: At the inorganic site, fertilizer was incorporated into the soil within a week of application. At the organic site, the inorganic fertilizer was 9‐18‐9 (May 27, 2020). At the inorganic site the inorganic fertilizer was 16‐15.3‐40 (April 9, 2019), 10‐34‐0 (May 12, 2020), and 10‐34‐0 (May 14, 2023).

### 2.2 Precipitation data

The study used the ATMOS‐41 microclimate sensor suite (METER Group, Inc.) to gather hourly precipitation data. Because ATMOS‐41 does not record snowfall or the snow–water equivalent, these data were obtained from the National Oceanic and Atmospheric Administration weather station at Adrian Lenawee County Airport, situated just under 20 km from the two sites.

### 2.3 Flow data collection

Low flow rates were measured using a sharp‐crested 45° V‐notch weir (Agri Drain Corp.; Shokrana & Ghane, 2021). Two conditions must be met when using the V‐notch to measure drainage discharge: (1) the flow must be less or equal to the weir's design capacity and (2) the water level in the discharge area must remain below the maximum height of the V‐notch. To ensure these conditions were monitored, a HYDROS‐21 water level sensor was used to record the hourly water level. However, during high‐flow conditions, when the weir capacity was exceeded or the water level rose above the V‐notch apex, a TIENET‐350 area–velocity sensor (Teledyne ISCO) installed downstream was used to measure hourly drainage discharge. By combining these two flow measurements, the hourly drainage discharge data for this study were obtained. Surface runoff was negligible at both sites; therefore, runoff losses were excluded from this analysis.

### 2.4 Phosphorus data collection

Water samples were collected year‐round using a Teledyne ISCO autosampler, following a daily composite sampling strategy that involved taking six aliquots per day (Dialameh & Ghane, 2022). These samples, stored in 1000‐mL plastic containers, were transported to the lab weekly for analysis. Upon collection, the water samples were filtered through a 0.45‐µm filter for DRP analysis. TP was analyzed in unfiltered water samples within 7 days using the alkaline persulfate digestion method (Patton & Kryskalla, 2003). The samples were analyzed using a Gallery Discrete Analyzer from Thermo‐Fisher‐Scientific, using colorimetric techniques.

### 2.5 Sensitivity analysis for tracking new P in agricultural fields

The sensitivity analysis was designed to distinguish the new P dataset from the legacy P dataset. Similar to Osterholz et al. (2024), three trackers were initiated when fertilizer was applied. Tracker A recorded the number of days since the application, Tracker B tracked the cumulative precipitation following the application, and Tracker C measured the annual cumulative new P period. The new P period refers to intervals characterized by relatively elevated P concentrations directly resulting from recent fertilizer applications.

Based on the sensitivity analysis, new P applications noticeably changed the flow concentration when applied fertilizer was ≥3 kg ha^−1^. It took approximately 120 days for the newly applied P to integrate into the soil and become part of the legacy P pool, whereas approximately 250 mm of precipitation was adequate to completely wash off the new P after fertilizer application. The operational conditions were structured around these trackers. If Tracker B recorded 250 mm of cumulative precipitation before Tracker A reached 120 days, Tracker C was stopped. Conversely, if 250 mm of precipitation was not reached before the 120‐day mark, all P in the soil was considered legacy P, and Tracker C was similarly stopped. In cases where a farmer reapplied fertilizer at rates >3 kg ha^−1^ before either Tracker A or Tracker B met the stop criteria, both trackers were reset to zero, and counting was started anew, with the new P days being added to the existing total in Tracker C. Additionally, if the year ended without either Tracker A or Tracker B reaching their respective stop criteria, the remaining new P depletion days and cumulative precipitation were carried over to the following year. In years when multiple fertilizer applications occurred with intervals exceeding the new P loss span (120 days), all independent P applications were considered to determine the cumulative new P period for that year.

### 2.6 Data analysis

#### 2.6.1 Regression model

All site data, including P load and discharge, underwent quality control procedures, including the removal of outliers and values below detection limits, to ensure data integrity and model reliability. Subsequently, hourly data were aggregated to a daily time step by summing values, while the daily mean was used for water table depth. Following the sensitivity analysis standards outlined in Section 2.5, the new P period was derived from the dataset. The remaining data, after this process, is referred to as the “legacy P dataset.” The dataset was randomly split into calibration and validation datasets to ensure that both were robust and exhibited similar data distribution to avoid over‐ or under‐fitting the datasets and gave priority to the best predictor (Figures S1 and S2; Breiman, 2001) and (Belgiu & Drăgu, 2016). The legacy P dataset was split into a 4:1 ratio, with 20% designated as the “legacy P validation set” and the remaining 80% as the “legacy P calibration set.”

The calibration dataset, which served as the model's calibration data, was fitted using the linear regression model, as described by Osterholz et al. (2023). This approach, adapted from the linear regression equation on Time, Discharge, and Season model by Hirsch et al. (2010), was chosen due to its strong data‐driven characteristics. Similar to Osterholz et al. (2023), we removed the time‐dependent function because the experiment spanned a relatively short period (6 years). The linear regression model is expressed as:

(1) $$\mathrm{In}\left(l\right)=\beta_{1}\;\mathrm{In}\left(Q\right)+\beta_{2}\sin\left(2\pi t\right)+\beta_{3}\cos\left(2\pi t\right)+\beta_{0}+\varepsilon$$

where *l* represents the P load (DRP or TP) through the drainage discharge (kg ha^−1^); *Q* is the drainage discharge (cm day^−1^); and *t* is the day of the year, expressed as a decimal between 0 and 1 (dimensionless). The coefficients *β*_0_ *, β*_1_ *, β*_2_, and *β*_3_ are the fitted values from the machine learning model, whereas *ε* accounts for the unexplained variation or drift between the actual and predicted data. The machine learning model was trained using the linear regression tool from the SciKit‐Learn (Sklearn) library in Python (https://scikit‐learn.org/stable/), with the resulting coefficients applied during data validation. The linear regression machine learning model, under supervised learning, captures how flow and timing influence P load through drainage discharge. It estimates the coefficients of the linear regression, which can then be used to predict P load based on drainage discharge and timing of the P loss.

#### 2.6.2 Determining new P contribution

To determine the cumulative new P contribution for TP or DRP for a given field, the machine learning–derived function was used to predict the daily legacy P loss during the new P period, referred to as the predicted legacy P. The cumulative new P contribution was then obtained by subtracting the sum of predicted legacy P from the sum of observed P loss during the new P period. Following Osterholz et al. (2023), the predicted legacy P data for all years of the experiment were aggregated to create a singular dataset. The new P contribution percentage in any time frame was calculated as the ratio of cumulative new P contribution for that period divided by the observed cumulative P load during the period, and the observed cumulative P load was the sum of daily P load (Section S2 in the Supporting Information). New P contributions were evaluated primarily from October through January, acknowledging that P losses following fertilizer application can persist for several months beyond the application date.

#### 2.6.3 Event and base flow separation

External factors such as fertilizer application, storm‐induced runoff, and site‐specific conditions (e.g., soil compaction, slope, macro pores, and water table depth) can introduce abrupt spikes or dilution in P concentration (Dialameh & Ghane, 2023; King, Williams, & Fausey, 2015; Kleinman et al., 2015). We also observed abrupt P concentration variations in our data, which introduced noise into the hybrid statistical‐machine learning approach. To address this issue, the calibration data for each field were split into event flow and base flow datasets (daily resolution), which were then trained separately using the machine learning algorithm. An event flow starts when the flow was above the mean base flow (82.5 percentile of flow or higher) and was greater than the initial flow measurement by 20% and ended when the flow returned to the mean base flow for 2 consecutive days. This resulted in two distinct equations, one for event flow and one for base flow, each with its own set of function coefficients. These coefficient pairs were then applied concurrently during validation to ensure that the full dynamics of the system were accurately represented. This approach, with the model's TP and DRP load predictions trained and analyzed in the natural log and then transformed to their original values, yielded a more accurate fit and eliminated event underestimation for all calibration and validation datasets (Figures S3 and S4).

#### 2.6.4 Statistics to evaluate the regression model

To evaluate the performance of the machine learning model in predicting the coefficients of the linear regression for predicting legacy P load, several statistical evaluation measures were applied to the calibration and validation datasets: (a) Kling–Gupta efficiency (KGE) test assessed the model's ability to capture variability and predictive accuracy; (b) Nash–Sutcliffe efficiency (NSE) test evaluated how well the model's predictions matched observed data relative to the mean; and (c) percent bias (PBIAS) measured the systematic bias in the model's predictions (Almeida & Coelho, 2023; Knoben et al., 2019; Moriasi et al., 2015). Additionally, a visual diagnostic was conducted to ensure that the observed and predicted trends were similar, confirming that the model performed as expected (Moriasi et al., 2007; Osterholz et al., 2024). The new P contributions in this analysis were evaluated on an annual basis. All new P periods were extracted from the yearly data to form a unified dataset that captured the new P contributions for each year.

For the P nutrient simulations, model performance was categorized into four classes: very good when NSE >0.65 or PBIAS ≤ ±10%, good when 0.50 ≤ NSE ≤ 0.65 or ±10% < PBIAS < ±15%, satisfactory when 0.35 < NSE < 0.50 or ±15% ≤ PBIAS < ±30%, and unsatisfactory when NSE ≤ 0.35 or PBIAS > ±30% (Moriasi et al., 2007, 2015). The KGE was classified as good when KGE ≥−0.41 and unsatisfactory when KGE <−0.41 (Knoben et al., 2019). It is important to note that the positive values of KGE indicate good model performance, and it is considered a more stringent measure of model performance compared with NSE (Knoben et al., 2019; Mathevet et al., 2023). The performance standard reflects a model's accuracy and reliability, and these criteria were used to thoroughly evaluate the model.

#### 2.6.5 New P confidence band

Similar to Osterholz et al. (2024), we used the 95% confidence interval (*α* = 0.05) to assess the significance and validity of the new P contribution to the P load. To conduct this analysis, we combined the calibration and validation datasets to form a larger dataset, from which the confidence interval was determined. The confidence band provided insight into the uncertainty of the model's predictions: a wider band indicated greater uncertainty, whereas a narrower band suggested less uncertainty. This band helps us visualize the potential range of the model's errors, and any data points falling outside the band suggest that an external factor may be influencing the change. For the new P contribution to be considered significant, it must be statistically different from zero, meaning the confidence interval should not intersect with zero. If the interval is less than or includes zero, the new P contribution is deemed immeasurable (with respect to the model's accuracy) or insignificant for that nutrient.

## 3 RESULT AND DISCUSSION

The organic site had seven fertilizer applications, and the inorganic site had three fertilizer applications (Table 1). At the organic site, most of the P fertilizer occurred late in the year (October–December), leading to some P days transferring to the following year. In the years 2021 and 2024, new P losses were observed at the organic site despite no P fertilizer was applied in those years.

### 3.1 Performance evaluation of the regression model

#### 3.1.1 Performance of predicting load

**FIGURE 2:**
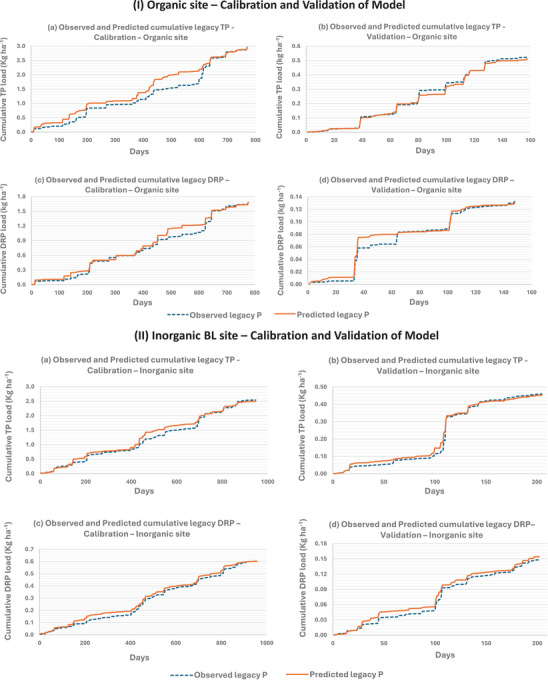
Predicted vs. observed cumulative legacy total phosphorus (TP) and dissolved reactive phosphorus (DRP) loads for organic (I) and inorganic (II) sites during calibration (a and c) and validation (b and d) periods. The *x*‐axis shows non‐continuous legacy days aggregated across 6 years as a result of the omitted new P periods. The new P periods are those with relatively higher P concentration induced by fertilizer applications.

The comparison of daily predicted and observed cumulative values for the calibration and validation datasets showed strong predictive accuracy with minimal bias (Figure 2). For legacy P predictions at the inorganic site, the difference between the observed and predicted TP load in calibration was 1.8% (2.541 kg vs. 2.496 kg), and in validation, it was 1.3% (0.460 kg vs. 0.454 kg). For DRP at the inorganic site, the difference between observed and predicted loads in calibration was 0.14% (0.602 kg vs. 0.601 kg), and in validation, it was 4.05% (0.148 kg vs. 0.154 kg). At the organic site, the difference between observed and predicted TP loads in calibration was 1.0% (2.931 kg vs. 2.963 kg), and in validation, it was 4.6% (0.538 kg vs. 0.513 kg). For DRP at the organic site, the difference between observed and predicted loads in calibration was 0.3% (1.685 kg vs. 1.680 kg), and in validation, it was 2.3% (0.133 kg vs. 0.130 kg).

The hybrid statistical‐machine learning model, developed for the regression equation, was trained and validated on a logarithmic scale, with predictions converted back to original values. The model demonstrated minimal bias, closely aligning predictions with observed data, and effectively captured variability across conditions. The statistical results in Table 2 confirm its strong performance in modeling load data variability, correlation, and bias.

**TABLE 2:** Model statistical evaluation measures for calibration, validation, and cumulative load data at the two on‐farm sites.

Site	Period	NSE		KGE		PBIAS (%)	
		TP load	DRP load	TP load	DRP load	TP load	DRP load
Organic	Calibration	0.70^vg^	0.76^vg^	0.72^vg^	0.73^vg^	0.83^vg^	2.00^vg^
	Validation	0.79^vg^	0.79^vg^	0.71^vg^	0.77^vg^	0.03^vg^	0.61^vg^
Inorganic	Calibration	0.71^vg^	0.70^vg^	0.71^vg^	0.84^vg^	−2.70^vg^	−3.10^vg^
	Validation	0.70^vg^	0.72^vg^	0.72^vg^	0.83^vg^	0.20^vg^	−1.30^vg^

Abbreviations: DRP, dissolved reactive phosphorus; KGE, Kling–Gupta efficiency; NSE, Nash–Sutcliffe efficiency; PBIAS, percent bias; TP, total phosphorus; vg, very good performance (Moriasi et al., 2015).

#### 3.1.2 Performance for predicting concentration

Although the model was not directly used to predict concentration, concentration was made the subject of the formula in the linear regression equation, as shown in Section S1 in the Supporting Information, with which the predicted concentration could be calculated. With an average KGE of 0.36, NSE of 0.25, and PBIAS of 6.82%, we can see that although there was room for improvement in performance, the hybrid statistical‐machine learning approach performed reasonably well with average moderate statistical values. The instances of reduced performance within this overall moderate range can be attributed to unexplained concentration spikes during intermittent flow events (Figure S5). Overall, the regression model demonstrated strong predictive capabilities with minimal bias and with good ability to capture both load and concentration variability.

### 3.2 Regression model coefficients

By distinguishing between event flow and base flow (Figure S6), our model could independently analyze and generate coefficients for each flow type (Table S2). This separation was crucial for reducing the influence of enrichment associated with abrupt changes in P loss behavior during high‐discharge events. Consequently, the machine learning model's performance improved substantially, allowing it to more accurately capture the underlying nutrient dynamics and generate more reliable predictions, as illustrated in the inorganic site results (Figures S5, S6, and S7). When event and base flows were modeled jointly (without separation), model performance metrics were NSE = 0.50, KGE = 0.25, and PBIAS = 40.99%. In contrast, after separating event and base flows, the performance improved to NSE = 0.64, KGE = 0.81, and PBIAS = 0.47%, demonstrating that flow separation enhanced model accuracy and reduced prediction bias. Upon completing the model calibration, the output consisted of two distinct sets of coefficients: one set represented the event flow, whereas the other corresponded to the base flow.

### 3.3 Observed P concentrations from the sites

We observed that the flow‐weighted mean P concentration in drainage discharge at the organic site was two to five times higher than that of the inorganic site during the study period (0.367 vs. 0.185 mg L^−^^1^ for TP and 0.281 vs. 0.053 mg L^−^^1^ for DRP; Table 3). The average daily P concentrations at the organic site was statistically greater than the inorganic site (*p*‐value < 0.01 for TP and DRP; Table S3). Given that both sites have comparable overall soil physical characteristics (clay loam subsoil and similar bulk density; Table S1), the primary reason for the elevated P concentration at the organic site was the greater P fertilizer rate (average annual of 48.2 kg ha^−1^ at the organic site and 23.2 kg ha^−1^ at the inorganic site) and higher soil test P at the organic site. In our study, the average Mehlich‐3 soil test P concentrations at 0–15 cm depth were 85.8 ppm at the organic site and 32.8 ppm at the inorganic site, likely explaining the higher P concentrations observed at the organic site. Similarly, Schoumans et al. (2014) and McDowell and Sharpley (2001) demonstrated that P concentrations leached from soil were strongly correlated with soil test P levels. Kamrath and Yuan (2023) reported a median DRP concentration of 2.10 mg L^−1^ from organic‐fertilized sites, while DRP concentration was 1.52 mg L^−1^ from inorganic‐fertilized sites in the Western Lake Erie Basin region of the United States. Overall, P concentrations in drainage discharge were consistently higher at the organic site compared to the inorganic site due to the greater P application rate and higher soil test P levels.

**TABLE 3:** Phosphorus application, drainage discharge, and contribution of new P to total phosphorus (TP) loss at two on‐farm sites over the 6‐year study period (2019–2024).

Site	P form	Observed cumulative P load (kg ha^−1^)	Observed average daily P load^a^ (kg ha^−1^)	New P contribution^b^ (%)	FWMC^c^ (mg L^−1^)	FWMC^c^ new P period (mg L^−1^)	Average annual P application rate (kg ha^−1^)	Observed daily discharge (cm)
Organic	TP	7.54	0.006a	16.3	0.367	0.617	48.2	0.119a
	DRP	4.90	0.004a	20.6	0.281	0.441		
Inorganic	TP	3.73	0.003b	2.9	0.185	0.257	23.2	0.147a
	DRP	1.05	0.001b	BDL	0.053	0.090		

*Note*: Within each column, similar letters indicate no statistical difference at α = 0.01 based on two‐sample t‐test.

Abbreviations: BDL, below detection limit; DRP, dissolved reactive phosphorus; FWMC, flow‐weighted mean concentration; TP, total phosphorus.

^a^ Daily P values were used because the two sites had unequal P application days (569 vs. 156).

^b^ This is calculated by dividing the cumulative new P load over the study period by the observed cumulative P load for the same period.

^c^ Flow‐weighted mean concentration (FWMC) is calculated by dividing the total load—obtained by summing the daily concentrations multiplied by daily flows—by the total discharge over the same period.

### 3.4 Observed cumulative P load from the sites

Organic site had approximately twice the cumulative observed TP loss (7.54 kg ha^−^^1^ vs. 3.73 kg ha^−^^1^) and nearly four times the cumulative DRP loss (4.90 kg ha^−^^1^ vs. 1.05 kg ha^−^^1^) compared to inorganic site (Table 3). This difference was statistically different when comparing the average daily P load or the organic site to the inorganic site (*p*‐value < 0.01 for both TP and DRP). As the observed average daily drainage discharge was not statistically different for the two sites (*p*‐value = 0.68; Table 3), this outcome can be explained by the statistically greater average daily P concentrations in drainage discharge observed at the organic site (Section 3.3), driven by the greater P fertilizer rate and higher soil test P at the organic site. Consistent with King, Williams, Macrae, et al. (2015), organic field sites generally exhibited higher P concentrations in the drainage discharge and, consequently, greater phosphorus losses than inorganic field sites. Overall, the organic site exhibited greater potential for P load than the inorganic site, underscoring the need for strategies to minimize P loss.

### 3.5 New P contributions from the sites

**FIGURE 3:**
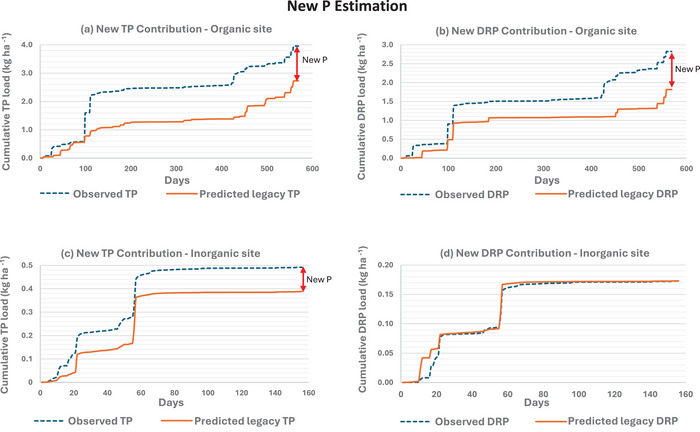
(a and b) Predicted legacy P for the new P period and observed P for the same period at the organic site. (c and d) Predicted legacy P for the new P period and the observed P for the same period at the inorganic site. The red lines depict the new P (TP or DRP) contribution. The days on the *x*‐axis are the number of new P days throughout the 6‐year experimental period.

The organic site had a substantial new P contribution, whereas the inorganic site had a minor new P contribution for TP and none for DRP (Figure 3 and Table 3). Specifically, the organic P sources accounted for 16% (1.228 kg ha^−1^) of the TP and 21% (1.009 kg ha^−1^) of the DRP losses during the new P period. In contrast, the inorganic P source contributed only 2.9% (0.104 kg ha^−1^) to TP loss and had no measurable DRP contribution. Statistical analysis confirmed these trends: the organic site's 95% confidence interval did not include zero (Figure S8a), indicating significant new P contributions for both TP and DRP. Conversely, the inorganic site's confidence interval for DRP intersects with zero (Figure S8b), suggesting not statistically significant new DRP contribution following fertilizer application. These findings align with Osterholz et al. (2024), who also reported greater new P loss from organic P sources than inorganic P sources. Overall, the organic site contributed more to new P loss than did the inorganic site.

One reason for the higher new P loss at the organic site compared to the inorganic site is the difference in fertilizer applications frequency (Tables 1 and 3; Brooker et al., 2024; Byers et al., 2025). Frequent P applications at the organic site led to elongated new P periods, and consequently a greater cumulative drainage discharge during the new P period (Figure S9). This increases the likelihood of new P coinciding with precipitation events, thereby increasing new P load (Osterholz et al., 2023, 2024). The organic site experienced 569 new P days (1218 legacy P days), far exceeding 156 new P days at the inorganic site (1261 legacy P days), due to more frequent fertilizer applications (seven at organic vs. three at inorganic). Osterholz et al. (2023) reported that extending the new P loss risk period up to 180 days increased the cumulative DRP loss in tile drainage by capturing more drainage discharge events. The other reason for the higher new P loss at the organic site is the greater flow‐weighted mean concentration of P during the new P period (0.617 mg L^−1^ vs. 0.257 mg L^−1^ for TP, and 0.441 mg L^−1^ vs. 0.09 mg L^−1^ for DRP; Section 3.3; Figure S9).

Conversely, the inorganic site, compared to the organic site, with fewer P applications, exhibited no measurable new DRP contributions and minimal new TP contributions (2.9%), attributed to lower number of new P days, leading to lower drainage discharge, and lower flow‐weighted mean concentration of P (Figure S8b). Notably, one of the three new P loss periods at the inorganic site involved only a single minor discharge event, resulting in negligible new P loss and contributing to the site's lower overall new P contributions. Overall, the greater cumulative drainage discharge during the new P period and higher flow‐weighted mean concentration of organic P led to greater new P loss contributions at the organic site compared to the inorganic site (Table 3).

### 3.6 Contribution of manure timing to new P loss at the organic site

**FIGURE 4:**
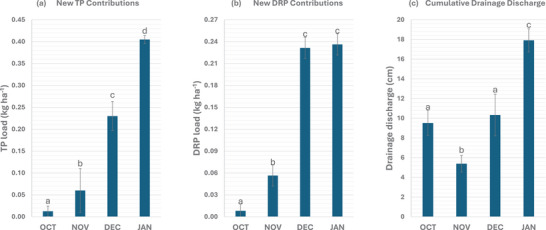
(a) New total phosphorus (TP), (b) dissolved reactive phosphorus (DRP) load, and (c) drainage discharge. Values for October and January represent two‐year averages. Values for November and December represent new P loss from that month in a single year. New TP and DRP contributions correspond to P application (manure) at the organic site: October (25.7 kg ha^−1^ for 2020 and 2022 cumulatively), November (24.9 kg ha^−1^ for 2023), December (5.3 kg ha^−1^ for 2019), and January (133.7 kg ha^−1^ for 2019 and 2022 cumulatively). The error bars represent standard error.

New P losses from December to January were greater than those from October to November (Figure 4a,b). Specifically, two‐month average new TP loss was 0.340 kg ha^−1^ from December to January, compared to 0.037 kg ha^−1^ from October to November. Similarly, two‐month average new DRP loss was 0.234 kg ha^−1^ from December to January, versus 0.032 kg ha^−1^ from October to November. Several factors can explain the increased P losses during the winter months: increased drainage discharge, more frequent freeze‐thaw cycles, dormant vegetation, and greater P application rate.

The first factor behind the higher New P loss in December–January was the increased drainage discharge compared to October–November (Figure 4c). Drainage discharge is widely recognized as the primary driver of nutrient transport (Gentry et al., 2007; Ghane et al., 2016; King, Williams, & Fausey, 2015; Pease et al., 2018; Van Esbroeck et al., 2016). A prolonged dry period in late summer and early fall often extended into October and November, reducing both drainage discharge and P loss during those months (Figure S10). Measurements at the organic site showed that declining precipitation in August and September dried the soil, lowered the water table, and stopped flow (Figure S11). As fall transitioned to winter, increasing precipitation raised the water table above the drain pipe, restoring flow and substantially increasing P loads.

Another factor that increased drainage discharge during winter and amplified P loss was the more frequent freeze‐thaw cycles (Hoffman et al., 2019; Seybold et al., 2022). Based on timelapse photography captured during winters of water years 2022–2025, the organic site experienced seven to nine snowmelt cycles per year (Table S4) that were greater than historical averages in southeast Michigan and sometimes these events were propagated by rain‐on‐snow occurrences. Isard and Schaetzl (1998) and Sinha and Cherkauer (2010) reported three to five cycles of freeze‐thaw on average for southeastern Michigan during years of 1951–1980. Our results show that freeze‐thaw cycles have increased in southeast Michigan compared to historical occurrences, increasing the risk of P loss. The data from Seybold et al. (2022) indicate that winter in the United States is warming more rapidly than any other season, reducing the frequency of freezing days. As a result, precipitation increasingly falls as rain atop existing snowpack, generating rain on snow and snowmelt‐driven runoff. These winter flows bypass dormant vegetation that do not uptake P and mobilize P that was previously immobilized in frozen soils.

Another factor affecting the greater new P loss during winter was the higher P fertilizer application rate during these months, which also contributed to greater new P loss compared to October and November. During December and January across all years, three P applications totaled 139 kg ha^−1^, while October and November had three applications totaling 50.6 kg ha^−1^ (Table 1). The disproportionate impact of winter applications is exemplified by a large manure application on January 5, 2022, which resulted in 53.8% of the total annual TP loss (88.7% as new TP) and 53.2% of the total annual DRP loss (87.7% as new DRP) occurring within 60 days of application. Forty‐four days after a January 5, 2022 manure application, two snowmelt events on February 17 and 22 accounted for roughly 20% of the year's TP loss. Furthermore, despite having 2.7‐folds increase in the entire P fertilizer application, December‐January resulted in over 9 times the new TP and over 7 times the DRP loss compared to October‐November. This disproportionate increase is largely attributed to higher drainage discharge during the winter months. Previous studies by Ballantine and Tanner (2013), Mandal et al. (2021), Ortega‐Pieck et al. (2020), and Smith et al. (2015) have shown that fertilizer applications in December and January significantly elevate both new and legacy P losses (Figure 4). Kokulan et al. (2022) reported that P leaching increased exponentially from January to March compared to October to December. Applying P immediately after harvest allows time for soil bonding (P sorption), which helps reduce leaching (Barrow, 2015; Notodarmojo et al., 1991).

Overall, the analysis confirmed that the onset of winter, characterized by increased drainage discharge, more frequent freeze‐thaw cycles, dormant vegetation, and greater P application rate, played a pivotal role in amplifying new P loss. These findings highlight the water quality benefits of applying manure shortly after harvest, when soils are drier and before winter begins, as this timing can help reduce the risk of P leaching during high‐flow winter conditions. In situations where subsurface injections are unavailable, incorporating manure into the soil using low disturbance tillage after surface broadcasting has been shown to be an effective strategy for reducing P loss (Daverede et al., 2004; Kleinman et al., 2022). This approach disrupts macropore connectivity and limits the direct transport of surface applied manure to tile drains.

### 3.7 Recommendations for reducing new P loss

To improve Lake Erie's water quality, both near‐term and long‐term P loss reduction strategies are needed. Brooker et al. (2024) and Byers et al. (2025) showed that fields with a high soil test P resulted in higher P loss in drainage discharge than field with lower soil test P. Thus, the first step is the soil test P drawdown by crop removal that addresses the legacy P loss, and this has shown to be a slow process (Gatiboni et al., 2025; Mott et al., 2025). For near‐term impact, nutrient management curbs new P loss and stabilizes soil test P near agronomic levels, supporting crop production and reducing fertilizer application rates (Kaiser et al., 2025; Kamrath & Yuan, 2023; Kleinman et al., 2022). Another near‐term impact strategy is water management, such as drainage water recycling, which reduces new and legacy P losses while providing supplemental irrigation (Hay et al., 2021). Controlled drainage shows promise but lacks sufficient research for effects on P loss compared to nitrate (Frankenberger et al., 2024).

## 4 SUMMARY AND CONCLUSIONS

Our study revealed key P dynamics in drainage discharge:

- The machine learning model accurately predicted both organic and inorganic TP load. Modeling event and base flows separately further improved performance.
- Flow‐weighted mean P concentrations in drainage discharge were two to five times higher at the organic site than the inorganic site, primarily due to greater application rate and higher soil test P levels. The organic site had higher cumulative and daily P losses than the inorganic site.
- The organic site had significantly higher new P loss compared with the inorganic site, with new TP contributing 16% versus 2.9% and DRP contributing 21% versus no measurable loss, respectively.
- Legacy P was the dominant form of P loss with 79%–100% contribution from both sites.
- Winter P applications (December‐January) led to substantially greater two‐month average new TP loss (0.340 kg ha^−1^) than fall applications (October‐November), which contributed just 0.037 kg ha^−1^ of TP. This was due to the increased drainage discharge, more frequent freeze‐thaw cycles, dormant vegetation, and greater P application rate that played a pivotal role in amplifying new P loss.

In conclusion, this study demonstrated the application of machine learning techniques to identify and quantify new sources of P contribution within the system at the field scale. To protect surface water quality, we recommend a combined strategy: soil P drawdown for long‐term reduction of legacy P loss, alongside nutrient and water management measures for near‐term mitigation of new P loss.

## AUTHOR CONTRIBUTIONS

**Emeka Aniekwensi**: Conceptualization; data curation; formal analysis; investigation; methodology; software; visualization; writing—original draft; writing—review and editing. **Ehsan Ghane**: Conceptualization; data curation; formal analysis; funding acquisition; investigation; methodology; project administration; resources; supervision; visualization; writing—original draft; writing—review and editing.

## CONFLICT OF INTEREST STATEMENT

The authors declare no conflicts of interest.

## Supporting information



Supplementary material includes additional tables and figures. The data for this article can be found in the Dryad link at: https://datadryad.org/dataset/doi:10.5061/dryad.hqbzkh1wm. The machine learning algorithm can be found at https://github.com/jorelix/New‐and‐Old‐Phosphorus‐modelling.git The event and base flow split tool can be found at can be found at https://event‐package‐website.web.app. The link to the Freeze‐Thaw Cycle video can be found at: https://www.youtube.com/watch?v=62LwsdOOMq4
