# Supplementary material for: Hybrid statistical‐machine learning approach for analyzing legacy and new phosphorus losses from subsurface drainage systems
Source: J Environ Qual. 2026 Feb 13;55(1):e70145. doi: 10.1002/jeq2.70145 (PMC12905519; doi:10.1002/jeq2.70145)
Supplement: Supplementary file 1 — Supplementary material includes additional tables and figures. The data for this article can be found in the Dryad link at: https://datadryad.org/dataset/doi:10.5061/dryad.hqbzkh1wm. The machine learning algorithm can be found at https://github.com/jorelix/New‐and‐Old‐Phosphorus‐modelling.git The event and base flow split tool can be found at can be found at https://event‐package‐website.web.app. The link to the Freeze‐Thaw Cycle video can be found at: https://www.youtube.com/watch?v=62LwsdOOMq4 [file JEQ2-55-0-s001.docx]

**Supplementary Material for**

**Hybrid Statistical**–**Machine Learning Approach for Analyzing Legacy and New Phosphorus Losses from Subsurface Drainage Systems**

Aniekwensi Emeka ^a^  Ehsan Ghane ^*, a^

^[a]^ Michigan State University, Department of Biosystems and Agricultural Engineering, East Lansing, 48823, MI, USA

Journal of Environmental Quality

* Corresponding Author: [ghane@msu.edu](mailto:ghane@msu.edu)

# Figure S1. Distribution of calibration and validation data set


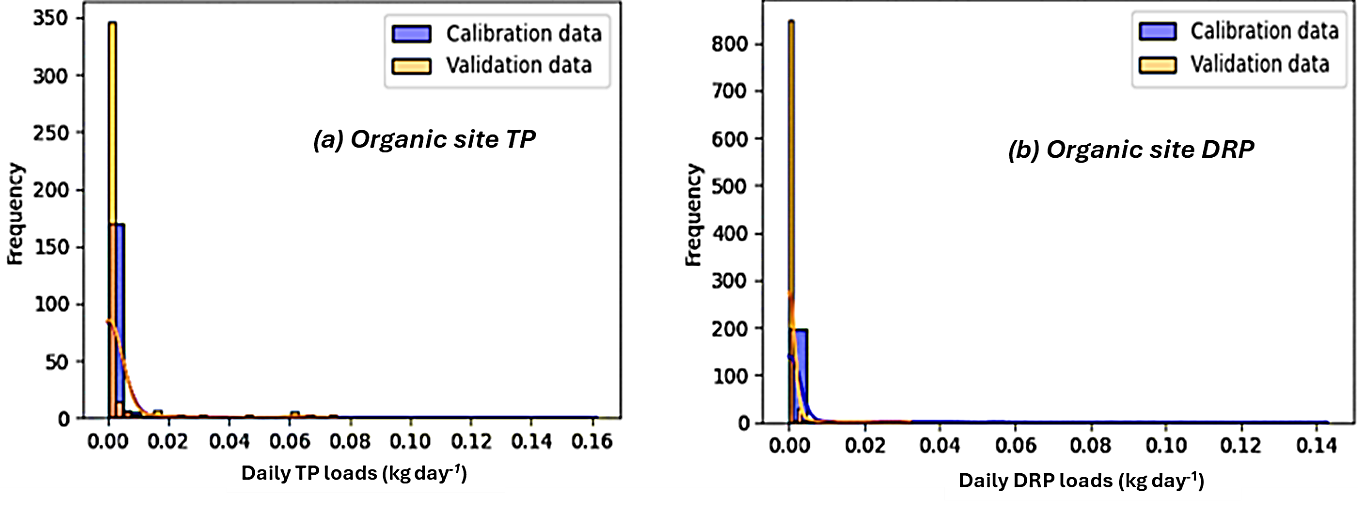


**Figure S1a.** (a and b) Organic site TP and DRP load for Calibration and Validation Data Distribution


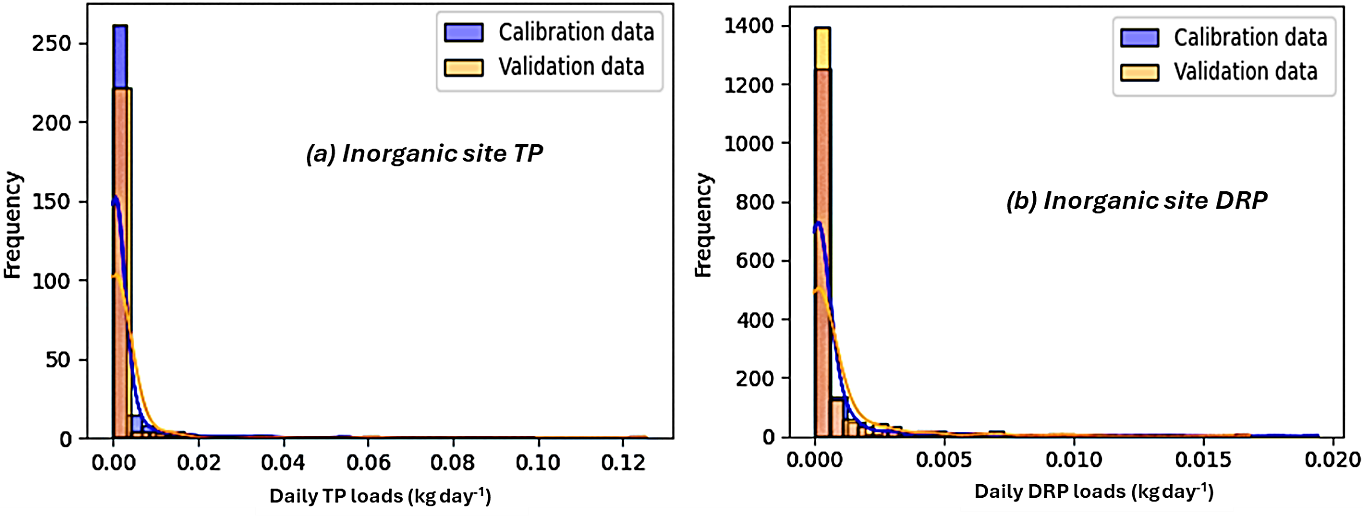


**Figure S1b.** (a and b) Inorganic site TP and DRP load for Calibration and Validation Data Distribution.

# Figure S2


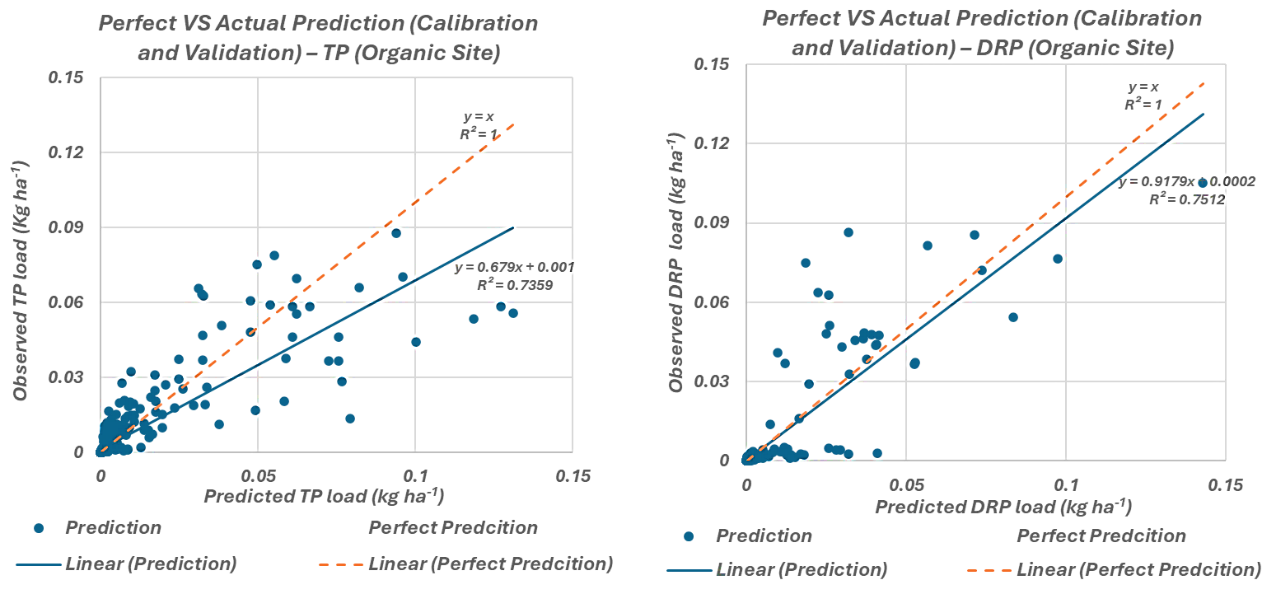


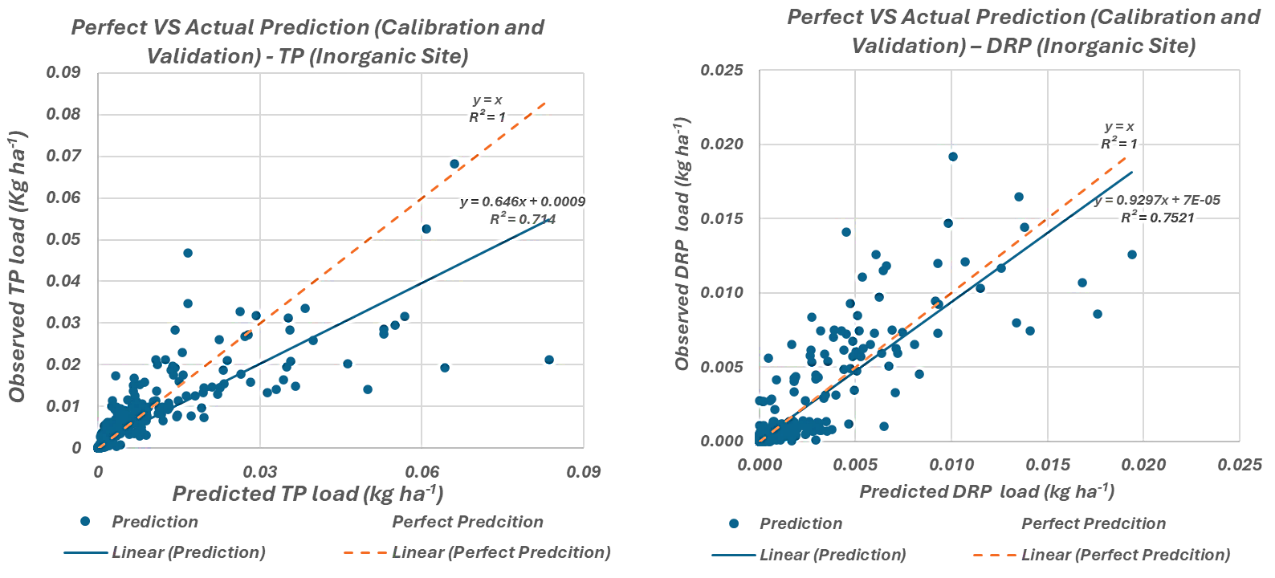


**Figure S2**. Comparing perfect prediction to actual prediction of the ML model.

# Figure S3


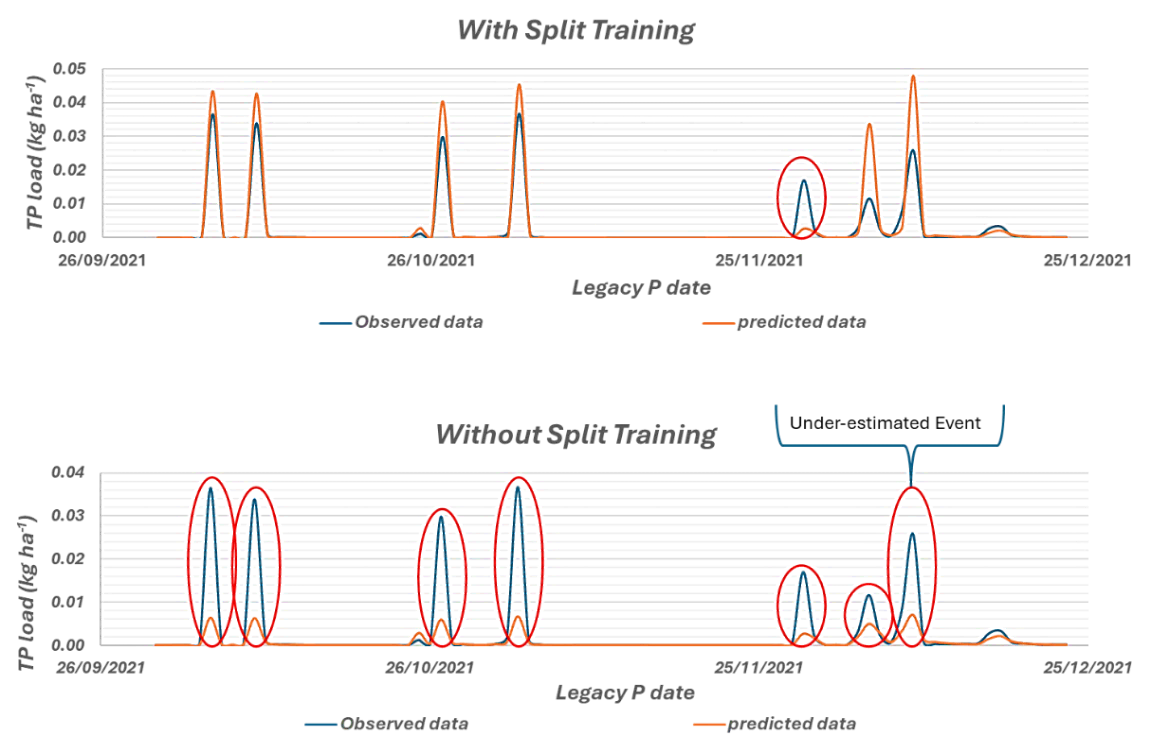


**Figure S3.** Excerpt of the dataset from the Inorganic site showing the effect of Event and Base flow Split on Model training. The red circles show event under estimation.

# Figure S4


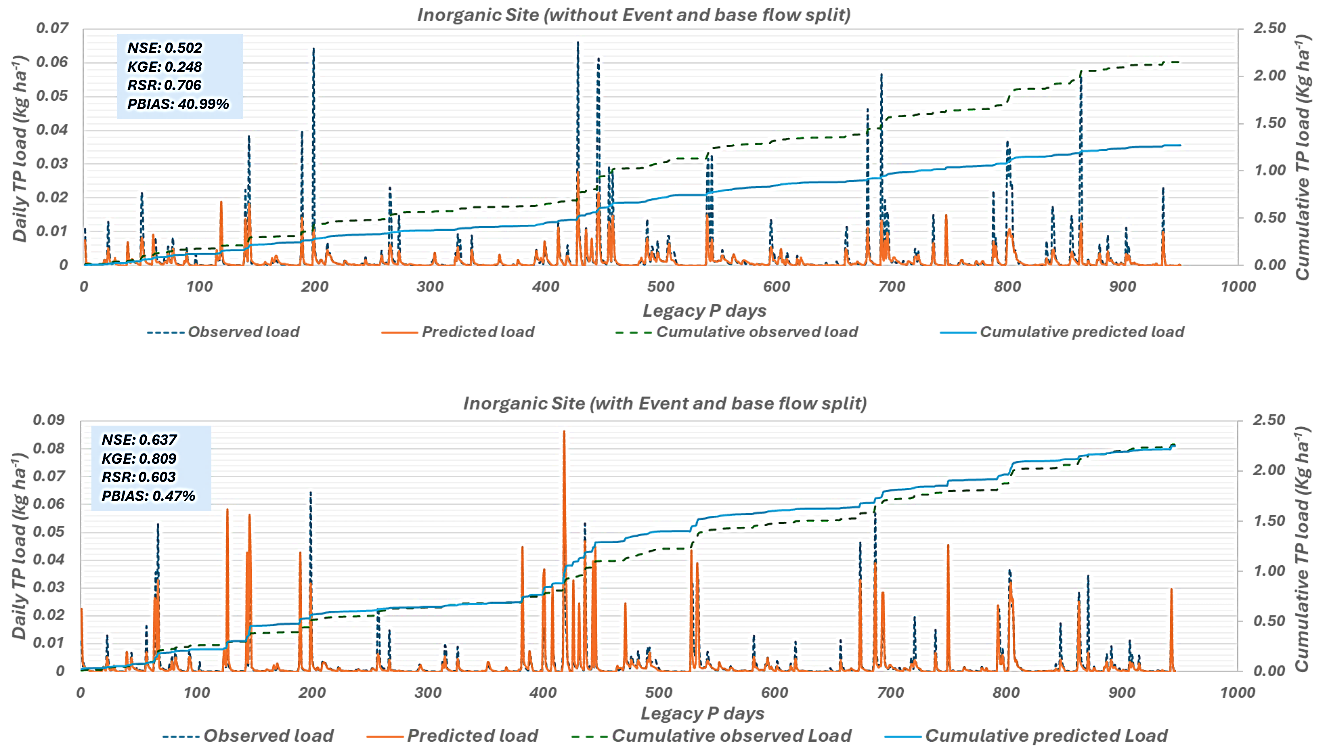


**Figure S4.** The difference in model performance resulting from Event and Base flow Split.

# Figure S5. Organic site


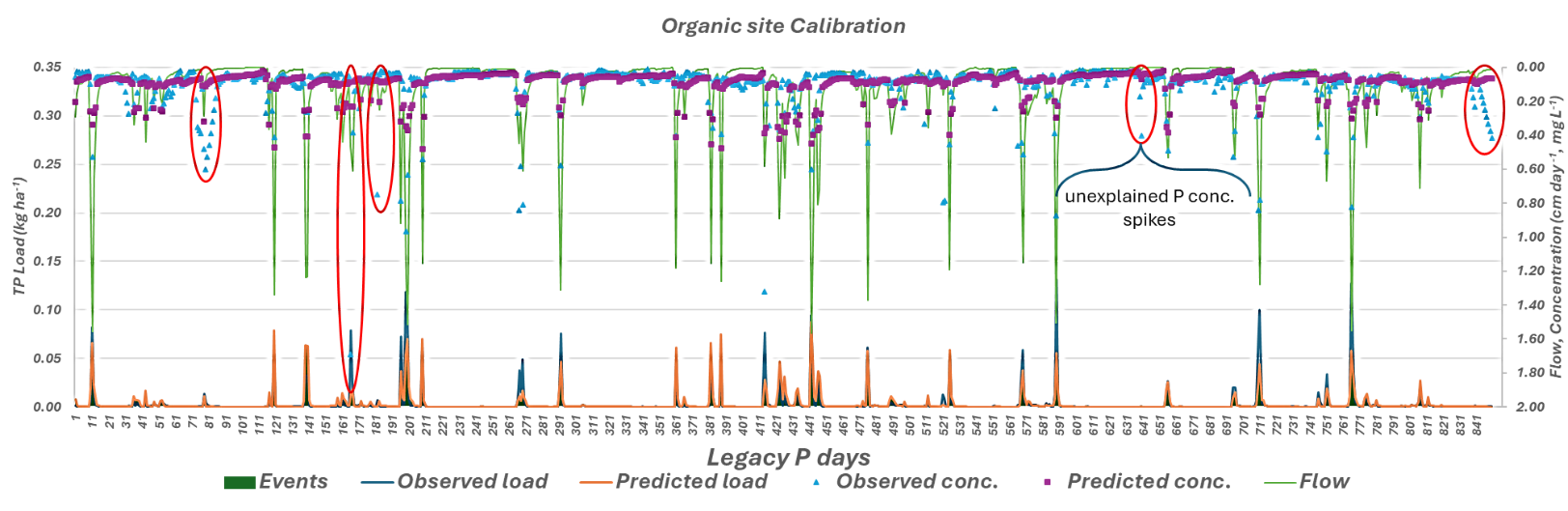


**Figure S5.** Calibration trend of Observed and Predicted TP Load and Concentration in the Organic site.

# Figure S6


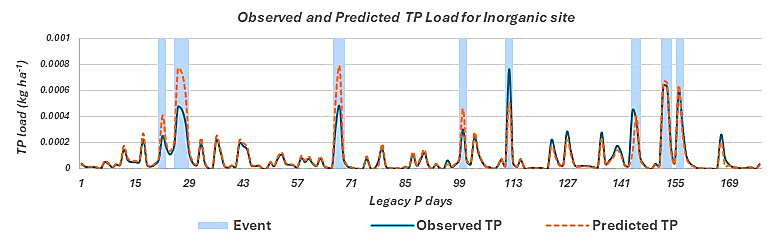


**Figure S6**. Plot of the predicted and observed legacy TP trends at the Inorganic site. The blue-shaded areas indicate the points where the event function was applied, and the unshaded areas represent the regions where the base flow function was utilized. Note: The days on the x-axis are non-continuous and are the combined legacy days from all six years of the experiment.

# Figure S7. Inorganic Site


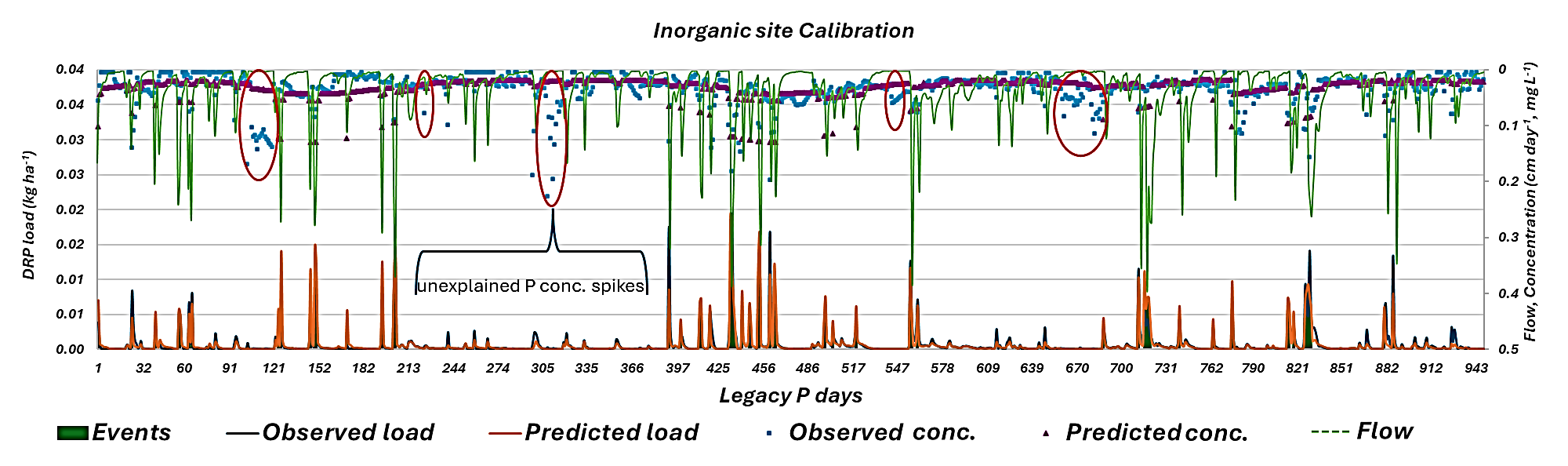


**Figure S7.** Calibration trend of Observed and Predicted TP Load and Concentration in the Inorganic site. *The days on the x-axis are the legacy P days at the inorganic site

# Figure S8


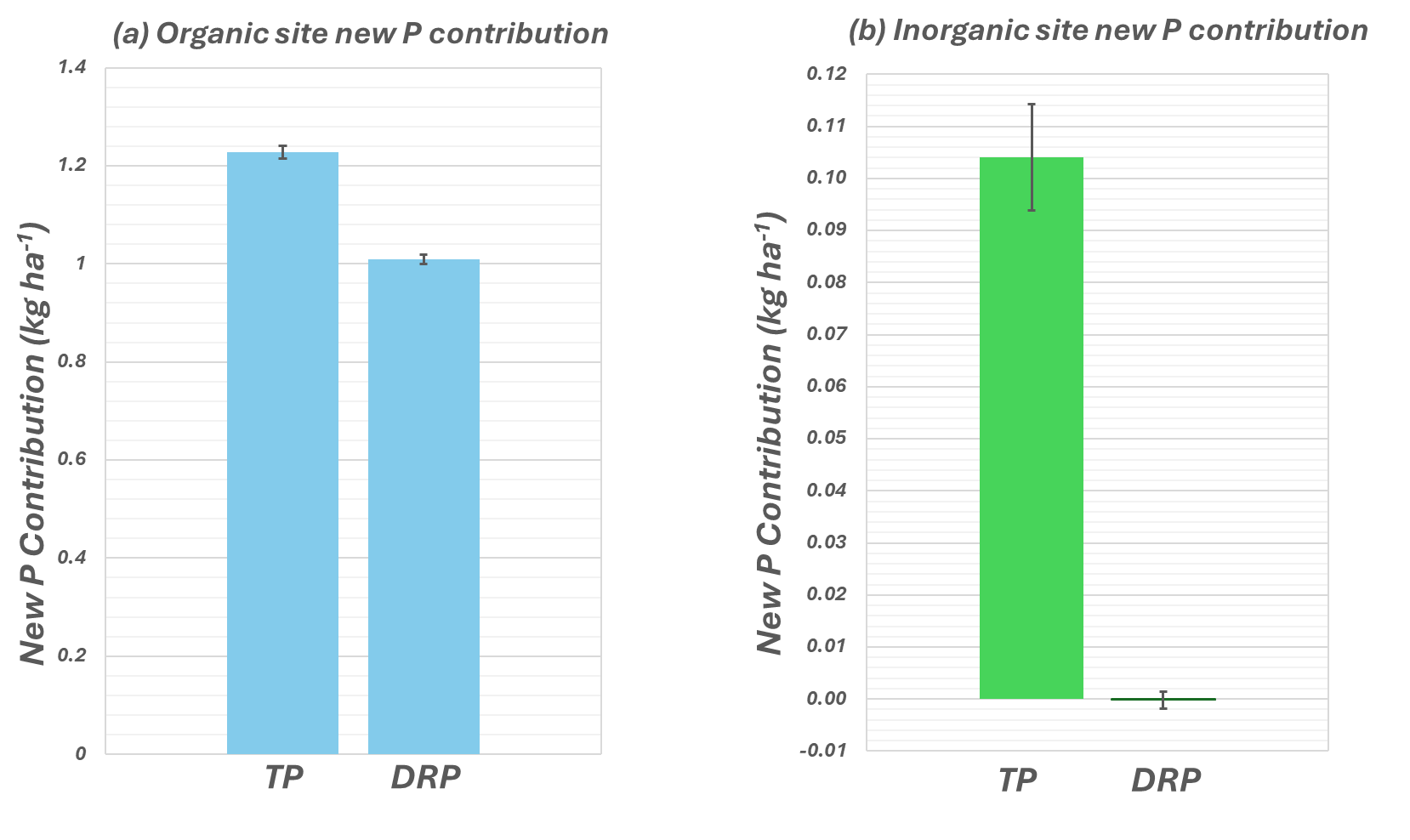


**Figure S8.** (a and b) Cumulative new P contributions of the Organic and Inorganic sites and the corresponding confidence intervals. If the confidence interval does not include zero, the new P loss is statistically significant. The whiskers show the 95% confidence interval of the new P contribution.

# Figure S9. New P Period, Drainage Discharge and P Loss


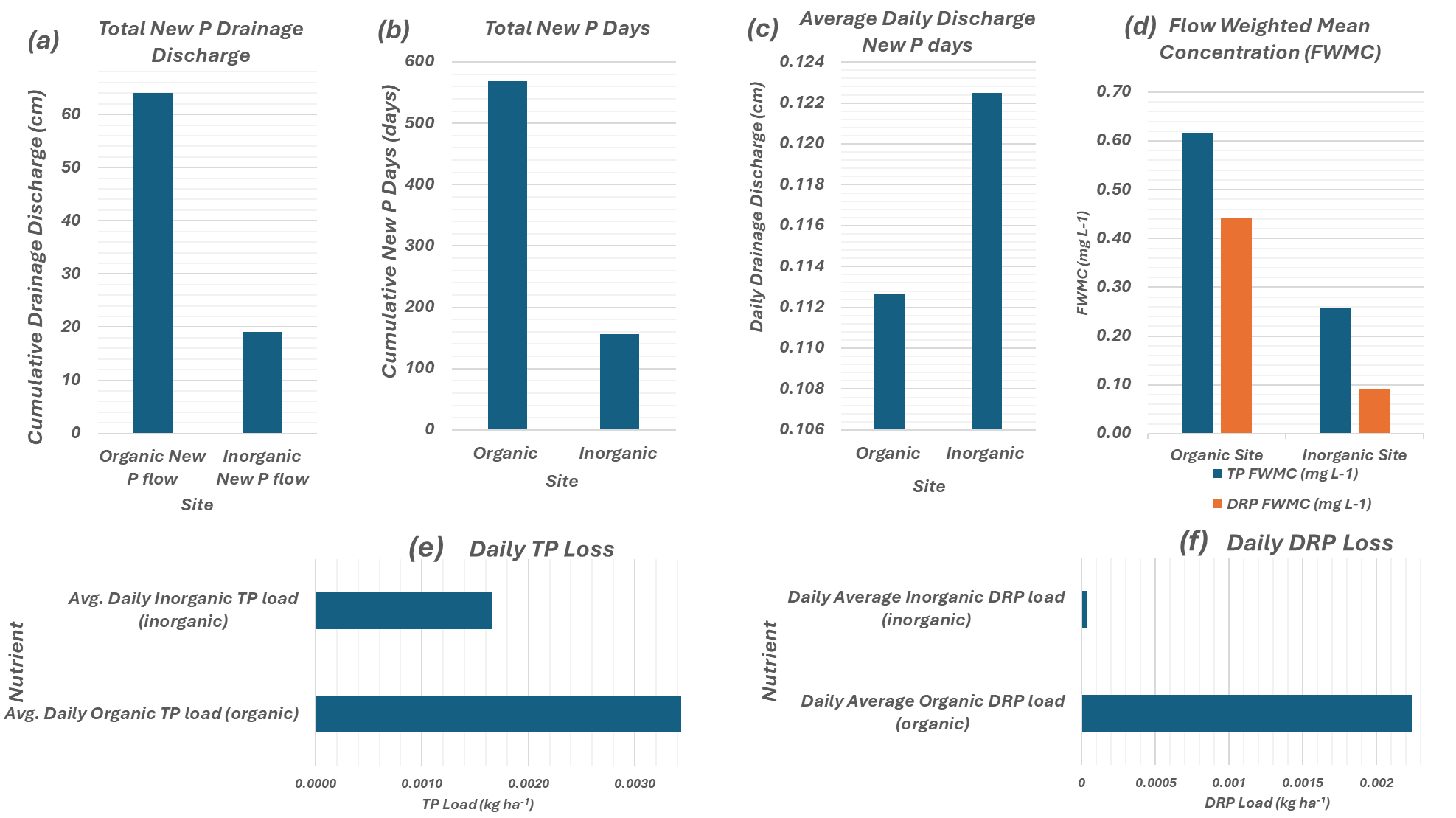


**Figure S9.** (a) Cumulative drainage discharge for the new P period during the study. (b) Number of new P days in the two fields. (c) Daily new P drainage discharge. (d) FWMC of the TP and DRP in both sites (e) daily TP load in both sites (f) Daily DRP load in both sites

# Figure S10. Date, Drainage Discharge, TP and DRP Loads – Organic Site


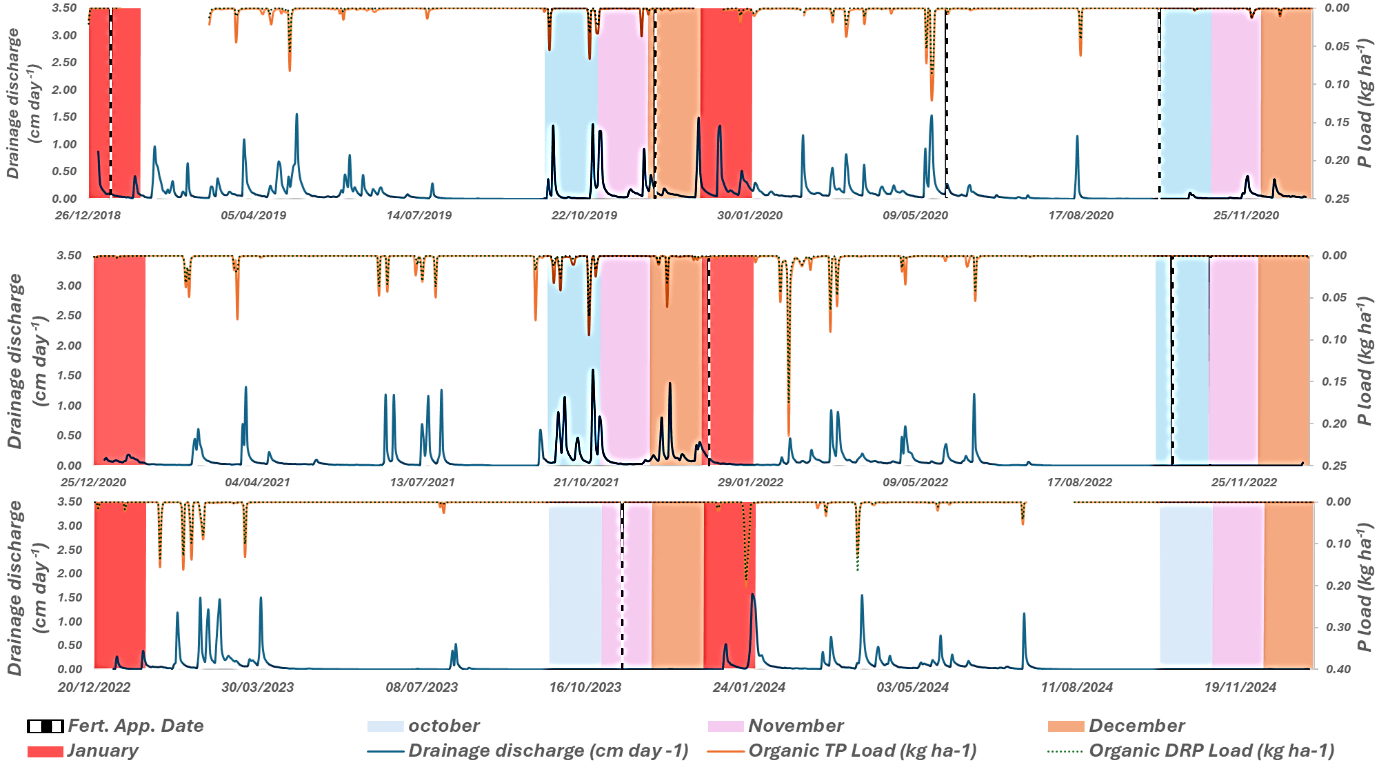


1. ***2019 - 2020***
2. ***2021- 2022***
3. ***2023- 2024***

**Figure S10**. Drainage discharge, TP and DRP Load at the Organic site during the 6 years experiment.

# Figure S11. Date, water level in structure and drainage discharge – Organic Site


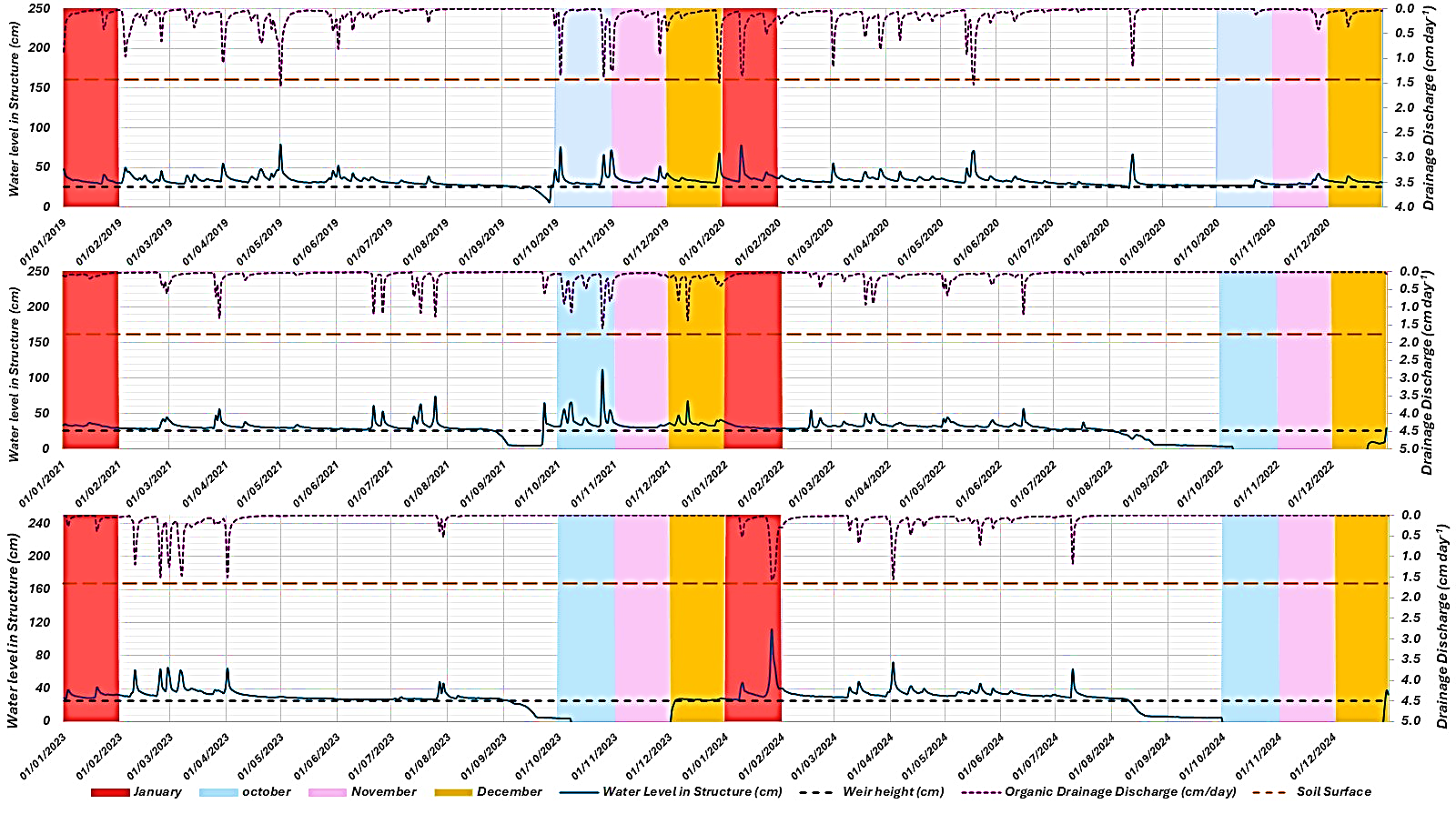


1. ***2019 - 2020***
2. ***2021- 2022***
3. ***2023 - 2024***

**Figure S11.** Drainage discharge, water level in structure at the Organic site over six years.

# Figure S12


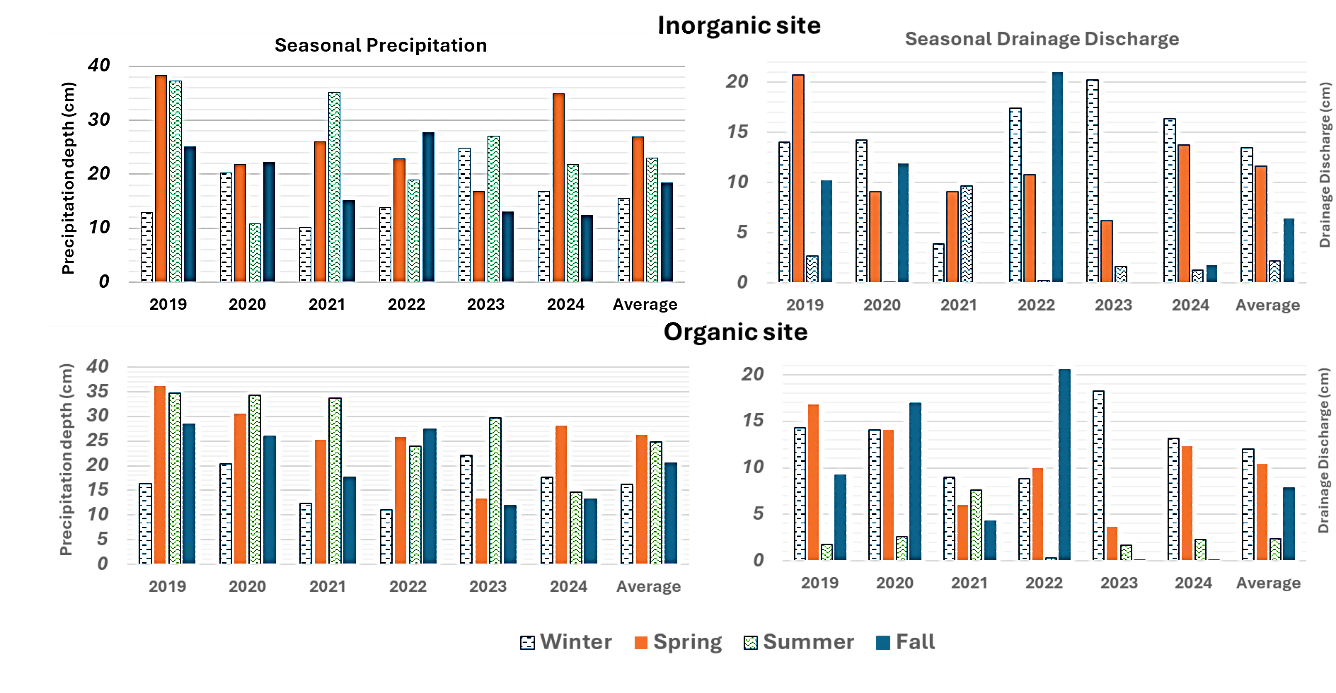


**Figure S12.** Precipitation and Drainage Discharge in the seasons of the year respectively.

# Figure S13


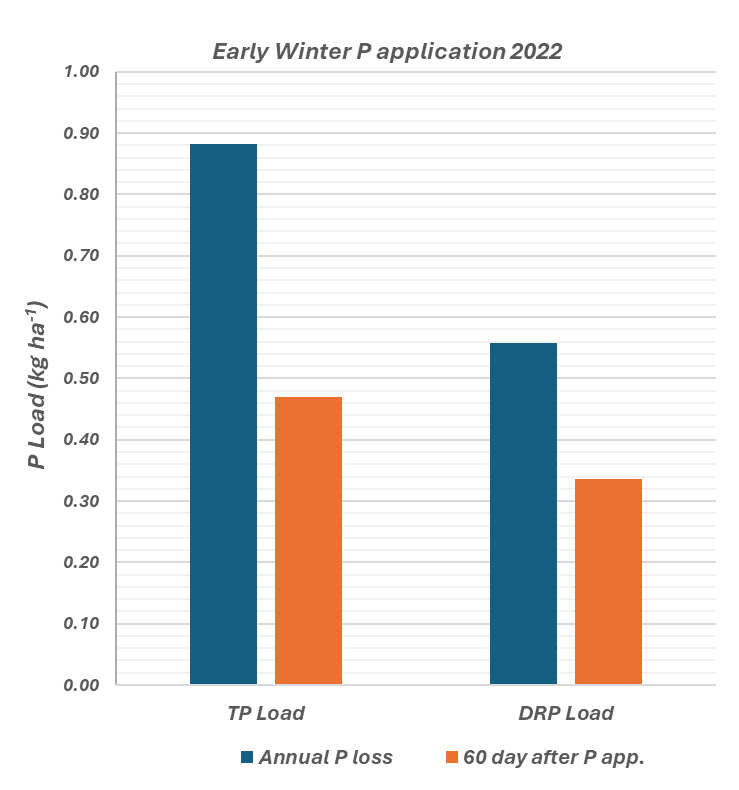


**Figure S12.** Extreme case of P loss following winter manure application (January 5, 2022) driven by elevated winter drainage discharge and freeze–thaw conditions.

# Figure S14


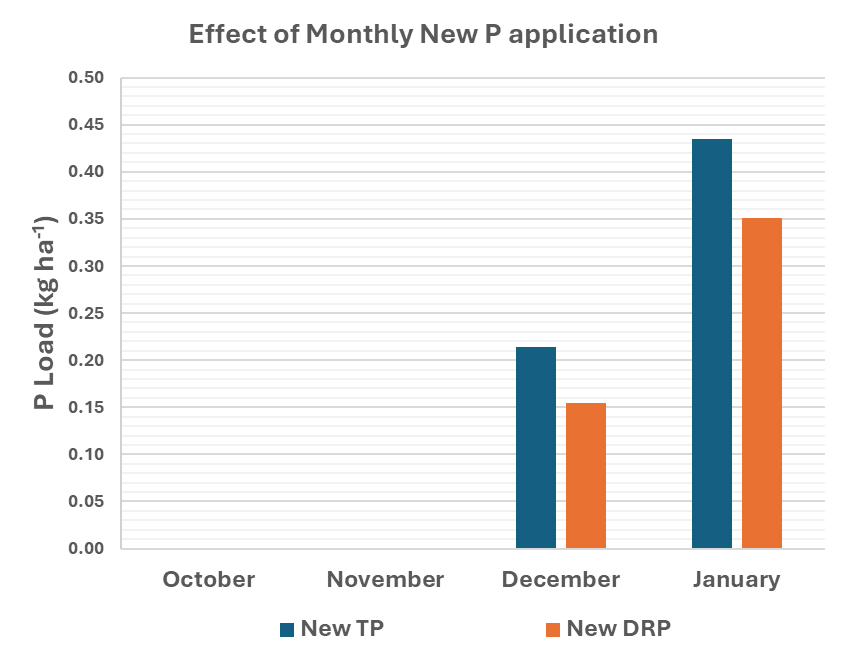


**Figure S13.** Each month represents the new P that was lost from the fertilizer application of that month over the course of the new P period (either 250 mm Precipitation or 120 days). Manure applications in October and November had no measurable new P loss.

# Table S1

**Table S1.** Sites soil properties.

| Sites | Depth  (cm) | Sand  (%) | Silt  (%) | Clay  (%) | Soil type | Bulk density  (g/cm^3^) | Organic matter (%) | Depth to restrictive layer  (cm) |
| --- | --- | --- | --- | --- | --- | --- | --- | --- |
|  | 0 – 23 | 53.7 | 25.1 | 21.2 | Sandy clay loam | 1.4 | 1.7 | 305 |
| Organic | 23 –76 | 41.5 | 29.1 | 29.4 | Clay loam | 1.5 | NA |  |
|  | 76 – 94 | 37.5 | 28.5 | 34.0 | Clay loam | 1.5 | NA |  |
|  | 94 – 200 | 22.0 | 45.0 | 33.0 | Clay loam | 1.9 | NA |  |
| Inorganic | 0 – 23 | 34.2 | 32.2 | 33.6 | Clay loam | 1.4 | 2.4 | 170 |
|  | 23 – 76 | 31.0 | 36.4 | 32.6 | Clay loam | 1.5 | NA |  |
|  | 76 – 102 | 28.0 | 38.9 | 33.1 | Clay loam | 1.5 | NA |  |
|  | 102 – 170 | 35.0 | 33.0 | 32.0 | Clay loam | 1.7 | NA |  |
|  | 170 – 203 | 34.0 | 37.0 | 29.0 | Clay loam | 1.9 | NA |  |

# Table S2

**Table S2**. WRDS coefficients for TP and DRP at the Organic and Inorganic sites.

| Coefficients | TP (Organic site) | | DRP (Organic site) | | TP (Inorganic site) | | DRP (Inorganic site) | |
| --- | --- | --- | --- | --- | --- | --- | --- | --- |
|  | Base Flow | Event flow | Base Flow | Event flow | Base Flow | Event flow | Base Flow | Event flow |
| β1 | 1.2762 | 3.4797 | 1.37 | 10.05 | 1.0883 | 3.8657 | 0.97 | 2.83 |
| β2 | −0.2526 | −0.6887 | 0.03 | 0.24 | −0.3449 | −0.12251 | −0.37 | −1.08 |
| β3 | −0.1865 | −0.5084 | 0.22 | 1.64 | −0.0134 | −0.05 | 0.14 | 0.41 |
| β0 | −4.1347 | −11.2732 | −5.17 | −37.96 | −4.4896 | −15.95 | −6.01 | −17.52 |

β1 – Drainage discharge (Q) coefficient,

β2 and β3 – Seasonality factor coefficients

β0 – Intercept

*Note: *The model was fitted in the log form.*

# Table S3

**Table S3.** Statistical comparison of observed daily flow, load and concentration between the organic and inorganic sites.

| Response variable |  | Daily Flow  (cm) | Daily  TP load  (kg ha^-1^) | Daily  DRP load  (kg ha^-1^) | Daily average TP concentration (mg L^-1^) | Daily average DRP concentration (mg L ^-1^) |
| --- | --- | --- | --- | --- | --- | --- |
| Observed values | Organic | 0.119^a^ | 0.006 ^a^ | 0.004 ^a^ | 0.156 ^a^ | 0.077 ^a^ |
|  | Inorganic | 0.147^a^ | 0.003 ^b^ | 0.001 ^b^ | 0.102 ^b^ | 0.035 ^b^ |
| Sample size n | Organic | 2192 | 2192 | 2192 | 2192 | 2192 |
|  | Inorganic | 2192 | 2192 | 2192 | 2192 | 2192 |
| *p*-value |  | 0.68 | < 0.01 | < 0.01 | < 0.01 | < 0.01 |

^*^ Within each column, same letter indicates no statistical difference at α= 0.01 based on two-sample t-test.

# Table S4

**Table S4.** Number of freeze-thaw events at the Organic site in the water years 2022 - 2025

| Water Year | Freeze-thaw count | Dates  (mm/dd/yyyy) |
| --- | --- | --- |
| 2022 | 1 | 11/15/2021 |
|  | 2 | 11/30/2021 |
|  | 3 | 12/01/2021 |
|  | 4 | 01/02/2022 |
|  | 5 | 01/16/2022 |
|  | 6 | 02/02/2022 |
|  | 7 | 02/17/2022 |
|  | 8 | 02/24/2022 |
|  | 9 | 02/28/2022 |
| 2023 | 1 | 11/21/2022 |
|  | 2 | 12/14/2022 |
|  | 3 | 12/20/2022 |
|  | 4 | 12/30/2022 |
|  | 5 | 01/11/2023 |
|  | 6 | 01/25/2023 |
|  | 7 | 02/10/2023 |
|  | 8 | 02/27/2023 |
| 2024 | 1 | 12/01/2023 |
|  | 2 | 01/08/2024 |
|  | 3 | 01/10/2024 |
|  | 4 | 01/26/2024 |
|  | 5 | 01/29/2024 |
|  | 6 | 02/02/2024 |
|  | 7 | 02/18/2024 |
| 2025 | 1 | 12/03/2024 |
|  | 2 | 12/16/2024 |
|  | 3 | 12/26/2025 |
|  | 4 | 01/03/2025 |
|  | 5 | 01/07/2025 |
|  | 6 | 01/31/2025 |
|  | 7 | 02/05/2025 |
|  | 8 | 02/12/2025 |
|  | 9 | 02/27/2025 |
| Average | 8 |  |

# Table S5

**Table S5**. Contribution of flow and load by Event at both sites.

|  | | Total | Event contribution | Percentage event contribution to total (%) |
| --- | --- | --- | --- | --- |
| Organic site | Flow (cm) | 202.451 | 172.455 | 0.85 |
|  | TP (kg ha^-1^) | 7.540 | 7.238 | 0.95 |
|  | DRP (kg ha^-1^) | 4.900 | 4.772 | 0.99 |
| Inorganic Site | Flow (cm) | 208.069 | 168.536 | 0.81 |
|  | TP (kg ha^-1^) | 3.73 | 3.320 | 0.89 |
|  | DRP (kg ha^-1^) | 1.05 | 1.030 | 0.98 |

# Table S6

**Table S6**. Average model statistical evaluation measures for concentration prediction at the two on-farm sites.

| Site | Evaluation measure | TP concentration | DRP concentration |
| --- | --- | --- | --- |
| Organic site | KGE | 0.35 ^vg^ | 0.56 ^vg^ |
|  | NSE | 0.28 ^u^ | 0.47 ^s^ |
|  | PBIAS (%) | 8.16 ^vg^ | 8.85 ^vg^ |
| Inorganic site | KGE | 0.23 ^vg^ | 0.31 ^vg^ |
|  | NSE | 0.15 ^u^ | 0.08 ^u^ |
|  | PBIAS (%) | 3.06 ^vg^ | 7.19 ^vg^ |

KGE= Kling–Gupta Efficiency, NSE= Nash–Sutcliffe Efficiency, PBIAS= percent bias, vg = very good performance, g = good performance, s = satisfactory performance, u = unsatisfactory performance.

# Section S1

$$\mathrm{In}\left( l \right)=\beta_{1}\mathrm{In}\left( Q \right)+\beta_{2}\sin\left( 2\pi t \right)+\beta_{3}\cos\left( 2\pi t \right)+ \beta_{0} + \varepsilon$$

where l represents the P load (DRP or TP) through the drainage discharge (kg ha-^1^), Q is the drainage discharge (cm day^-1^), and t is the day of the year, expressed as a decimal between 0 and 1 (dimensionless). The coefficients β_0_, β_1_, β_2_, and β_3_ are the fitted values from the machine learning model, while ε accounts for the unexplained variation or drift between the actual and predicted data.

Assuming error = 0

$$\mathrm{In}\left( l \right)=\beta_{1}\mathrm{In}\left( Q \right)+\beta_{2}\sin\left( 2\pi t \right)+\beta_{3}\cos\left( 2\pi t \right)+ \beta_{0}$$

But $Q=$ $\frac{l}{c}$

Making c the subject of the formula we have

$$c= \frac{l}{exp(\frac{\mathrm{In}\left( l \right)-\sin\left( 2\pi t \right)-\beta_{3}\cos\left( 2\pi t \right)}{\beta_{1}})}$$

This equation counters the sinusoidal effect of the function and is used to calculate the concentration from the predicted load.

# Section S2

To determine the cumulative new P contribution (CNPC) (TP or DRP) for a given field, the machine learning-derived function was used to predict the daily legacy P loss during the new P period, referred to as the predicted legacy P for the new P period (PLPN). The total ENPC was then computed by subtracting the sum of PLPN from the observed P loss during the new P period (OPN).

$CNPC= \sum(OPN) - \sum(PLPN)$ (2)

where OPN is observed P in the new P loss period (kg ha^-1^), PLPN is the predicted legacy P in the new P period (kg ha^-1^), and CNPC is the cumulative new P contribution to P loads (kg ha^-1^).

The new P contribution percentage (NPCP) in any time frame was calculated as:

$NPCP= \frac{\mathrm{CNPC}}{\mathrm{COPL}} \times100$ (3)

$COPL = \sum(DPL)$ (4)

where NPCP is the new P contribution percentage (%), CNPC is the cumulative new P contribution (kg ha^-1^), COPL is the cumulative observed phosphorus load that can be TP or DRP (kg ha^-1^), and DPL is the daily observed phosphorus load, which can be TP or DRP (kg ha^-1^).

Github repo: <https://github.com/jorelix/New-and-Old-Phosphorus-modelling.git>
